# Machine learning for the prediction of volume responsiveness in patients with oliguric acute kidney injury in critical care

**DOI:** 10.1186/s13054-019-2411-z

**Published:** 2019-04-08

**Authors:** Zhongheng Zhang, Kwok M. Ho, Yucai Hong

**Affiliations:** 1 Department of Emergency Medicine, Sir Run Run Shaw Hospital, Zhejiang University School of Medicine, No. 3, East Qingchun Road, Hangzhou, 310016 Zhejiang Province China; 2 School of Population and Global Health, University of Western Australia, Perth, Australia

**Keywords:** Acute kidney injury, Critical care, Extreme gradient boosting, Urine output, Predictive modeling

## Abstract

**Background and objectives:**

Excess fluid balance in acute kidney injury (AKI) may be harmful, and conversely, some patients may respond to fluid challenges. This study aimed to develop a prediction model that can be used to differentiate between volume-responsive (VR) and volume-unresponsive (VU) AKI.

**Methods:**

AKI patients with urine output < 0.5 ml/kg/h for the first 6 h after ICU admission and fluid intake > 5 l in the following 6 h in the US-based critical care database (Medical Information Mart for Intensive Care (MIMIC-III)) were considered. Patients who received diuretics and renal replacement on day 1 were excluded. Two predictive models, using either machine learning extreme gradient boosting (XGBoost) or logistic regression, were developed to predict urine output > 0.65 ml/kg/h during 18 h succeeding the initial 6 h for assessing oliguria. Established models were assessed by using out-of-sample validation. The whole sample was split into training and testing samples by the ratio of 3:1.

**Main results:**

Of the 6682 patients included in the analysis, 2456 (36.8%) patients were volume responsive with an increase in urine output after receiving > 5 l fluid. Urinary creatinine, blood urea nitrogen (BUN), age, and albumin were the important predictors of VR. The machine learning XGBoost model outperformed the traditional logistic regression model in differentiating between the VR and VU groups (AU-ROC, 0.860; 95% CI, 0.842 to 0.878 vs. 0.728; 95% CI 0.703 to 0.753, respectively).

**Conclusions:**

The XGBoost model was able to differentiate between patients who would and would not respond to fluid intake in urine output better than a traditional logistic regression model. This result suggests that machine learning techniques have the potential to improve the development and validation of predictive modeling in critical care research.

## Background

Acute kidney injury (AKI) is common in the intensive care unit (ICU), and there is evidence that even a small increase in serum creatinine may be associated with increased risk of mortality [1–3]. AKI can be defined by either an elevation in serum creatinine or a reduced urine output according to the Kidney Disease: Improving Global Outcomes (KDIGO) guidelines [4]. Oliguric AKI constitutes a substantial proportion of the overall AKI population, and it imposes a great challenge for fluid management. Pathophysiologically, oliguria may represent an adaptive response in AKI, and once an effective circulatory volume is restored by positive fluid balance, urine output would improve. Under this circumstance, fluid administration or positive fluid balance can be considered beneficial and those who improve with more fluid can be considered as having volume-responsive (VR) AKI.

Intravenous fluid challenges are often used in critical care to restore blood pressure to improve urine output in patients with hypotension and oliguria, respectively [5, 6]. Recent epidemiological evidence, however, suggested that large positive fluid balance may not be useful to improve urine output in many patients with AKI and can even be harmful in worsening renal function through a number of possible mechanisms including excessive kidney edema [7]. This latter type of AKI can be considered as volume-unresponsive (VU) AKI [8]. Because early improvement in organ dysfunction is associated with an improved survival [9], it would be best if we can adjust the fluid treatment strategy by identifying which patients are having VR or VU AKI.

In animal models, fractional excretion of electrolytes was found to perform well in early differentiation between VR- and VU-AKI [10]. However, these promising results could not be replicated in human studies. Legrand et al. investigated the discrimination of urinary sodium to distinguish volume responsiveness, and it was found to have limited predictive value [11]. Currently, there is little clinical information on how we can identify patients with AKI who are VR and VU in terms of urinary output response. We hypothesized that advanced machine learning techniques may be useful to identify the most important clinical factors that can differentiate between patients with VR and VU AKI. In this study, we aimed to use machine learning techniques to develop and validate an AKI fluid-responsiveness model, called extreme gradient boosting (XGBoost), and compared the performance of this model to a conventional logistic regression model.

## Methods

### Source of data

A large US-based critical care database named Medical Information Mart for Intensive Care (MIMIC-III) was analyzed [12]. The MIMIC-III database is an integrated, de-identified, comprehensive clinical dataset containing all the patients admitted to the ICUs of Beth Israel Deaconess Medical Center in Boston, MA, from June 1, 2001, to October 31, 2012. There were 53,423 distinct hospital admissions for adult patients (aged 16 years or above) admitted to the ICUs during the study period. Since the study was an analysis of a third-party anonymized publicly available database with pre-existing institutional review board (IRB) approval, IRB approval from our institution was exempted. The study was reported according to the recommendations of the Transparent Reporting of a multivariable prediction model for Individual Prognosis Or Diagnosis (TRIPOD) statement [13].

### Participants

**Fig. 1:**
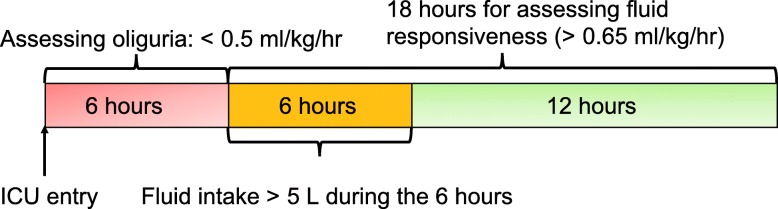
Schematic illustration of the time windows for the definition of oliguria and fluid responsiveness. Oliguria was defined as urine output < 0.5 ml/kg/h for the first 6 h after ICU admission. Fluid responsiveness was defined as urine output > 0.65 ml/kg/h for 18 h after initiation of fluid loading. It is noted that the time window for the definition of oliguria preceded the exposure of fluid input

Patient eligibility was considered when urine output was less than 0.5 ml/kg/h for the first 6 h after ICU admission. This definition was consistent with the urine output component of the KDIGO criteria [4]. To examine the impact of fluid resuscitation on subsequent urine output response, only patients with substantial fluid intake (> 5 l) within 6 h following the initial 6 h for assessing oliguria were eligible (Fig. 1). Fluid intake and urine output were extracted from the nursing chart system, which more accurately reflected the actual volume intake or output than the medical order system. Patients who left ICU during the observation period were excluded. Furthermore, patients receiving any diuretics and/or renal replacement therapy (RRT) on day 1 were excluded.

### Outcome (volume responsiveness)

The urine output within an 18-h period following the initial 6 h for defining oliguria was used as the outcome. Patients were considered as VR-AKI if he/she had urine output greater than 0.65 ml/kg/h, corresponding to a 30% increase as compared with the baseline value. Otherwise, they were defined as VU-AKI.

### Predictors of VR-AKI

Routinely collected clinical and laboratory variables obtained within the first 6 h of ICU admission were assessed for their ability to predict volume responsiveness. For some variables with multiple measurements, both the maximum and minimum values were assessed. Age, gender, ethnicity, admission type, elective surgery, type of ICU and presence of infection, and vital signs including respiratory rate, blood pressure, heart rate, and temperature were analyzed. In addition, laboratory data including glucose, white blood cell count (WBC), hematocrit, chloride, potassium, sodium, lactate, creatinine, blood urea nitrogen (BUN), coagulation profile, PaO2, PaCO2, and pH were included.

Because this was a hypothesis-generating epidemiological study, no attempt was made to estimate the sample size of the study; instead, all eligible patients in the database were included to maximize the statistical power of the predictive model. Because missing data may create bias, variables with > 70% missing values were excluded from further analysis. Other variables with a lesser degree of missing values were analyzed using multiple imputation method [14].

### Statistical analysis

Clinical characteristics between VR-AKI and VU-AKI groups were compared using either Student *t* test or rank-sum test as appropriate. Chi-square test or Fisher’s exact test was employed to compare the differences of the categorical variables [15]. A stepwise logistic regression model was used to select variables which were predictive of volume responsiveness. Both forward selection and backward elimination were used, testing at each step for variables to be included or excluded. Akaike Information Criterion (AIC) was used as the selection criteria to eliminate the predictors [16].

Extreme gradient boosting (XGBoost) combined with decision trees was employed to predict VR versus VU. A classification tree was used as the weak learner, and the learning objective function was binary logistic. The boosting method works by iteratively refitting a weak classifier (decision tree) to residuals of previous models. Each successive classifier focused more on misclassified observations during the previous round of fitting [17]. In this study, we employed 300 rounds of iterations for cross-validation process, which were expected to result in a powerful ensemble classifier with superior predictive accuracy. Overfitting can be a major problem in using machine learning techniques. The ability to understand the complex relationship in data while avoiding overfitting requires fine-tuned hyperparameters. XGBoost hyperparameters included learning rate, minimum loss reduction required to make a further partition on a leaf node of the tree, maximum depth of a tree, subsample ratio of the training instance. The original dataset was randomly partitioned into 5 equal-sized subsamples for bootstrap validation (BV). Specifically, 4 subsamples were used to train the model, which was then validated in the remaining 1 subsample. Hyperparameters were considered to be sufficiently tuned if (1) the BV training log-loss decreased as the number of trees increased and (2) BV testing log-loss was less than 0.693 and only slightly greater than the training log-loss (e.g., a log-loss of 0.693 is the performance of a binary classifier that performs no better than chance: − log 0.5 ≈ 0.693). We used a loop function (grid search) to select the hyperparameters that the minimum training log-loss should be greater than the 85th percentile, and the minimum testing log-loss should be less than the 8th percentile. After choosing the hyperparameters, the BV process was run for 100 times to determine the number of trees required for the final model. The number of trees in the final XGBoost model was determined by the minimum BV testing log-loss in each of the last 100 BV iterations and computed the 5th percentile of that distribution. This was a conservative approach to the selection of the number of trees, which was also described in the literature [18].

## Results

### Participants

**Fig. 2:**
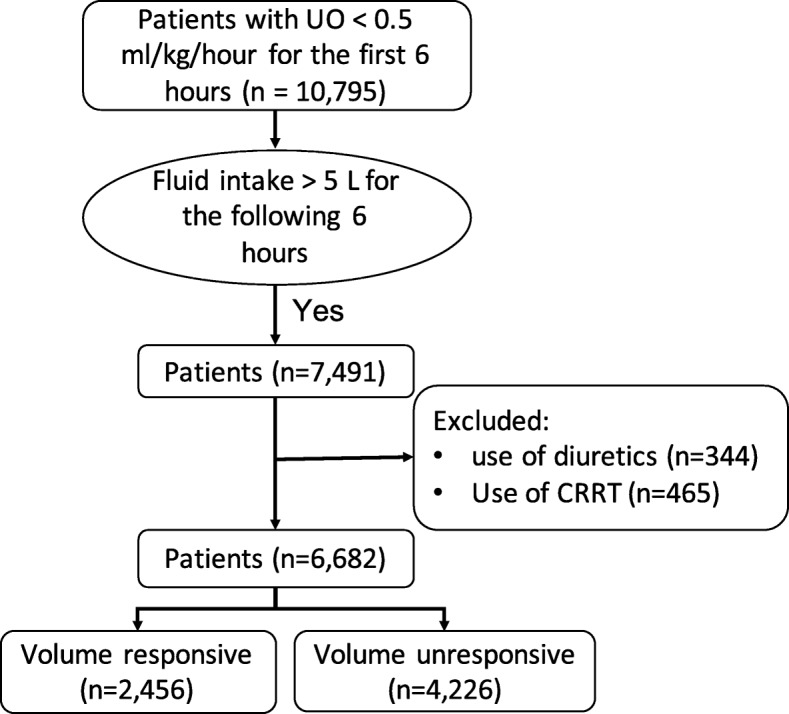
Flow chart of patient selection

Of the 10,795 patients with urine output < 0.5 ml/kg/h for the first 6 h after ICU admission, 7491 patients (69.4%) received fluid intake > 5 l within the following 6 h. A number of 809 patients were excluded because they received diuretics and/or RRT on the first day. A total of 6682 patients were included in our analysis; 2456 patients had VR-AKI, and 4226 patients had VU-AKI on day 1 in ICU (Fig. 2).

The differences in characteristics between VR and VU groups are described in Table 1. VR group had more patients of elective surgery prior to ICU admission than the VU group (18.2% vs. 13.6%; *p* < 0.001). The maximum serum creatinine concentration (1.74 ± 1.50 vs. 1.26 ± 0.98 μmol/l; *p* < 0.001) was higher, and the minimum bicarbonate concentration (20.82 ± 5.41 vs. 22.24 ± 4.32 mmol/l; *p* < 0.001) and maximum hematocrit (35.14 ± 5.69 vs. 35.93 ± 5.79%; *p* < 0.001) were lower in the VU group. The VR group had higher urinary pH (5.78 ± 0.86 vs. 5.66 ± 0.79; *p* < 0.001), lower urinary creatinine (111.61 ± 73.01 vs. 132.51 ± 79.89 mg/dl; *p* < 0.001), higher systolic blood pressure (89.16 ± 16.46 vs. 85.09 ± 18.03 mmHg; *p* < 0.001), higher albumin (3.03 ± 0.63 vs. 2.83 ± 0.66; *p* < 0.001), lower rate of mechanical ventilation (31.3% vs. 42.7%; *p* < 0.001), vasopressor use (22.8% vs. 30.0%; *p* < 0.001), and infection (47.6% vs. 62.4%; *p* < 0.001) than the VU group (Table 1).

**Table 1:** Characteristics between fluid responsive and non-responsive groups

Variables	Volume unresponsive (*n* = 4226)	Volume responsive (*n* = 2456)	*p* value
Demographic variables			
Gender male, *n* (%)	2203 (52.1)	1495 (60.9)	< 0.001
Age (mean (SD))	68.81 (15.14)	63.82 (16.67)	< 0.001
Ethnicity, *n* (%)			0.013
Asian	58 (1.4)	45 (1.8)	
Black	351 (8.3)	197 (8.0)	
Hispanic	94 (2.2)	83 (3.4)	
Unknown	517 (12.2)	326 (13.3)	
White	3206 (75.9)	1805 (73.5)	
Admission type (%)			< 0.001
Elective	656 (15.5)	485 (19.7)	
Emergency	3454 (81.7)	1908 (77.7)	
Urgent	116 (2.7)	63 (2.6)	
Elective surgery, *n* (%)	574 (13.6)	448 (18.2)	< 0.001
ICU type (%)			< 0.001
CCU	391 (9.3)	180 (7.3)	
CSRU	619 (14.6)	646 (26.3)	
MICU	1939 (45.9)	932 (37.9)	
SICU	771 (18.2)	385 (15.7)	
TSICU	506 (12.0)	313 (12.7)	
Vasopressor, *n* (%)	1266 (30.0)	561 (22.8)	< 0.001
Infection, *n* (%)	2639 (62.4)	1168 (47.6)	< 0.001
Mechanical ventilation, *n* (%)	1804 (42.7)	768 (31.3)	< 0.001
Serum laboratory variables, mean (SD) if not otherwise specified			
Serum creatinine (μmol/l)	1.74 (1.50)	1.26 (0.98)	< 0.001
Maximum glucose (mg/dl)	186.32 (99.16)	180.38 (85.71)	0.013
Minimum bicarbonate (mmol/l)	20.82 (5.41)	22.24 (4.32)	< 0.001
Maximum bilirubin (mg/dl, median [IQR])	0.80 [0.50, 1.90]	0.70 [0.40, 1.30]	< 0.001
Maximum bicarbonate (mmol/l)	23.80 (4.94)	24.88 (3.97)	< 0.001
Minimum chloride (mmol/l)	102.93 (6.65)	102.63 (6.16)	0.062
Maximum chloride (mmol/l)	107.94 (6.69)	108.28 (5.85)	0.036
Minimum hematocrit (%)	28.76 (5.98)	28.46 (6.21)	0.052
Maximum hematocrit (%)	35.14 (5.69)	35.93 (5.79)	< 0.001
Maximum lactate (mmol/l)	3.30 (2.85)	2.75 (1.90)	< 0.001
Minimum platelet (×10^9^/l, median [IQR])	181.00 [120.25, 253.75]	178.00 [128.00, 240.00]	0.479
Maximum potassium (mmol/l)	4.80 (0.94)	4.78 (0.96)	0.444
Maximum aPTT (median [IQR])	34.20 [28.42, 47.80]	32.50 [27.50, 40.60]	< 0.001
Maximum INR	1.89 (1.82)	1.59 (1.02)	< 0.001
Minimum sodium (mmol/l)	136.42 (5.92)	136.23 (5.34)	0.210
Maximum sodium (mmol/l)	140.20 (5.57)	140.23 (4.42)	0.850
Maximum BUN (median [IQR])	28.00 [19.00, 45.00]	20.00 [14.00, 30.00]	< 0.001
Minimum WBC (×10^9^/l)	11.67 (7.68)	10.61 (6.48)	< 0.001
Maximum WBC (×10^9^/l)	15.77 (10.08)	14.62 (9.69)	< 0.001
Minimum albumin (g/dl)	2.83 (0.66)	3.03 (0.63)	< 0.001
Vital signs, mean (SD)			
Maximum heart rate (/min)	106.76 (22.48)	105.97 (19.98)	0.147
Minimum systolic BP (mmHg)	85.09 (18.03)	89.16 (16.46)	< 0.001
Minimum diastolic BP (mmHg)	40.38 (11.69)	43.60 (10.97)	< 0.001
Maximum respiratory rate (/min)	28.17 (6.75)	27.52 (6.57)	< 0.001
Maximum temperature (°C)	37.48 (0.88)	37.64 (0.76)	< 0.001
Urinary biomarkers, mean (SD)			
Urinary pH	5.66 (0.79)	5.78 (0.86)	< 0.001
Urinary creatinine (mg/dl)	132.51 (79.89)	111.61 (73.01)	< 0.001

*Abbreviations*: *ICU* intensive care unit, *BP* blood pressure, *CCU* coronary artery unit, *CSRU* cardiac surgery recovery unit, *MICU* medical ICU, *SICU* surgical ICU, *TSICU* trauma-neuro surgical ICU, *SD* standard deviation, *IQR* interquartile range, *pH* potential hydrogen, *aPTT* activated partial thromboplastin time, *WBC* white blood cell count

### The stepwise logistic regression model

The results of stepwise logistic regression model are shown in Table 2. As expected, advanced age (odds ratio [OR] for a 20-year increase, 0.69; 95% confidence interval [CI], 0.63 to 0.75), a higher serum creatinine (OR for each 0.1 mg/dl increase, 0.98; 95% CI, 0.97 to 0.99), and lactate (OR for each 1-mmol/l increase, 0.91; 95% CI, 0.88 to 0.94) were associated with decreased probability of volume responsiveness. On the contrary, a greater value of albumin (OR for each 1-g/dl increase, 1.53; 95% CI, 1.38 to 1.71), systolic BP (OR for each 20-mmHg increase, 1.13; 95% CI, 1.04 to 1.23), and minimum bicarbonate (OR for each 5-mmol/l increase, 1.12; 95% CI, 1.02 to 1.22) were associated with increased probability of volume responsiveness (Table 2).

**Table 2:** Multivariable logistic regression model with stepwise variable selection

Variables	OR (95% CI)	*p* value
Gender (female as reference)	1.56 [1.36, 1.78]	< 0.001
Ethnicity (Asian as reference)		
Black	0.85 [0.49, 1.50]	0.574
Hispanic	1.09 [0.57, 2.10]	0.785
Unknown	0.88 [0.51, 1.52]	0.632
White	0.74 [0.44, 1.26]	0.261
ICU type (CCU as reference)		
CSRU	1.39 [1.04, 1.85]	0.027
MICU	0.94 [0.72, 1.22]	0.630
SICU	0.88 [0.66, 1.18]	0.391
TSICU	0.82 [0.60, 1.11]	0.200
Infection	0.77 [0.67, 0.89]	< 0.001
Mechanical ventilation	0.71 [0.61, 0.81]	< 0.001
Bilirubin (with every unit increment)	0.97 [0.95, 0.98]	< 0.001
Lactate (with every unit increment)	0.91 [0.88, 0.94]	< 0.001
Albumin (with every unit increment)	1.53 [1.38, 1.70]	< 0.001
Temperature (with every unit increment)	1.19 [1.10, 1.30]	< 0.001
Urinary pH (with every unit increment)	1.05 [0.97, 1.13]	0.252
Age (with each 20 years increment)	0.69 [0.63, 0.75]	< 0.001
Serum creatinine (with every 0.1 mg/dl increment)	0.98 [0.97, 0.99]	< 0.001
Maximum chloride (with every 20 mmol/l increment)	2.94 [2.08, 4.16]	< 0.001
Minimum chloride (with every 20 mmol/l increment)	0.44 [0.31, 0.60]	< 0.001
Maximum glucose (with every 20 mmol/l increment)	0.99 [0.97, 1.00]	0.164
Minimum bicarbonate (with every 5 mmol/l increment)	1.12 [1.02, 1.22]	0.014
Minimum hematocrit (with every 5% increment)	0.91 [0.86, 0.97]	0.002
BUN (with every 10 mmol/l increment)	0.94 [0.90, 0.99]	0.017
Maximum heart rate (with every 10 beats/min increment)	1.03 [1.00, 1.06]	0.095
Minimum systolic BP (with every 20 mmHg increment)	1.13 [1.04, 1.23]	0.003
Urinary creatinine (with every 50 mg/dl increment)	0.71 [0.68, 0.75]	< 0.001
Maximum aPTT (for every 10 s increment)	0.96 [0.93, 0.98]	< 0.001

An OR value greater than 1 indicates that the presence of a variable or increase in a continuous variable is associated with higher probability of volume responsiveness

*Abbreviations*: *OR* odds ratio, *BP* blood pressure, *aPTT* activated partial thromboplastin time, *BUN* blood urea nitrogen, *ICU* intensive care unit, *BP* blood pressure, *CCU* coronary artery unit, *CSRU* cardiac surgery recovery unit, *MICU* medical ICU, *SICU* surgical ICU, *TSICU* trauma-neuro surgical ICU

### The XGBoost model

**Fig. 3:**
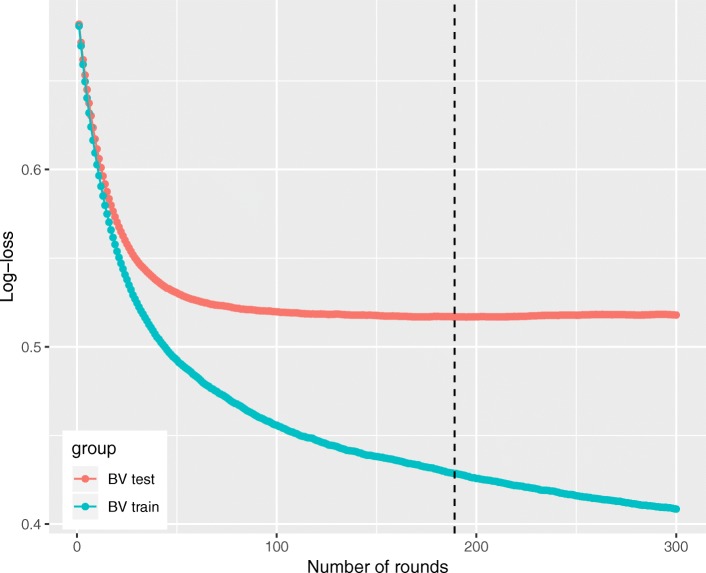
Training process of the extreme gradient boosting machine. Sample output of bootstrap validation (BV) during XGBoost hyperparameter tuning, using the values specified in the final XGBoost model (learning rate = 0.04, minimum loss reduction = 10, maximum tree depth = 9, subsample = 0.6, and number of trees = 300). Log-loss value for the training and testing datasets is shown in the vertical axis. The dashed vertical line indicates the number of rounds with the minimum log-loss in the test sample. The conditions of well-tuned model were satisfied: BV training log-loss decreases as the number of trees in an ensemble increases, and BV testing log-loss is less than 0.693 (e.g., a log-loss of 0.693 is the performance of a binary classifier that performs no better than chance: − log 0.5 ≈ 0.693) and only slightly more than BV training log-loss as the tree grows.

**Fig. 4:**
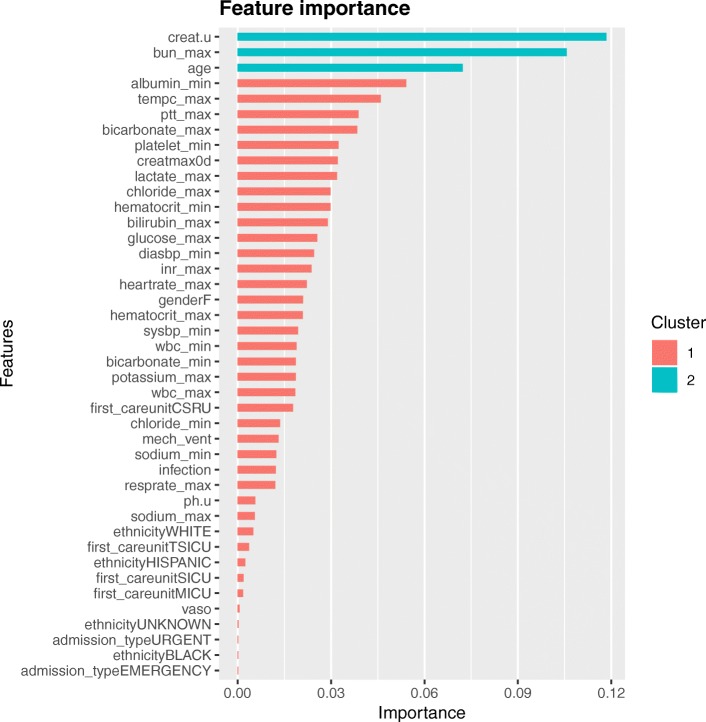
Feature importance derived from XGBoost model. Abbreviations and annotations: creat.u, urinary creatinine; bun_max, maximum blood urea nitrogen; creatmax0d, maximum creatinine on the day of ICU admission; diasbp_min, minimum diastolic blood pressure; inr_max, maximum international normalized ratio; heartrate_max, maximum heart rate; sysbp_min, minimum systolic blood pressure; first_careunitCSRU, first care unit is cardiac surgery recovery unit; mech_vent, mechanical ventilation; ph.u, urinary pH; TSICU, trauma-neuro surgical ICU; vaso, vasopressor

The hyperparameters used in our analysis were as follows (determine by grid search): learning rate = 0.04, minimum loss reduction = 10, maximum tree depth = 9, subsample = 0.6, and number of trees = 300. With these hyperparameters, bootstrap validation (BV) training log-loss decreases as the number of trees in an ensemble increases, and the BV testing log-loss was less than 0.693 and only slightly more than BV training log-loss as the tree grows (Fig. 3). Feature importance was calculated by the sum of the decrease in error when split by a variable, which reflects the contribution each variable makes in classifying VR versus VU. The urinary creatinine was the most important variable to distinguish VR and VU group, followed by maximum BUN, age, albumin, and maximum temperature (Fig. 4).

### Model performance

**Fig. 5:**
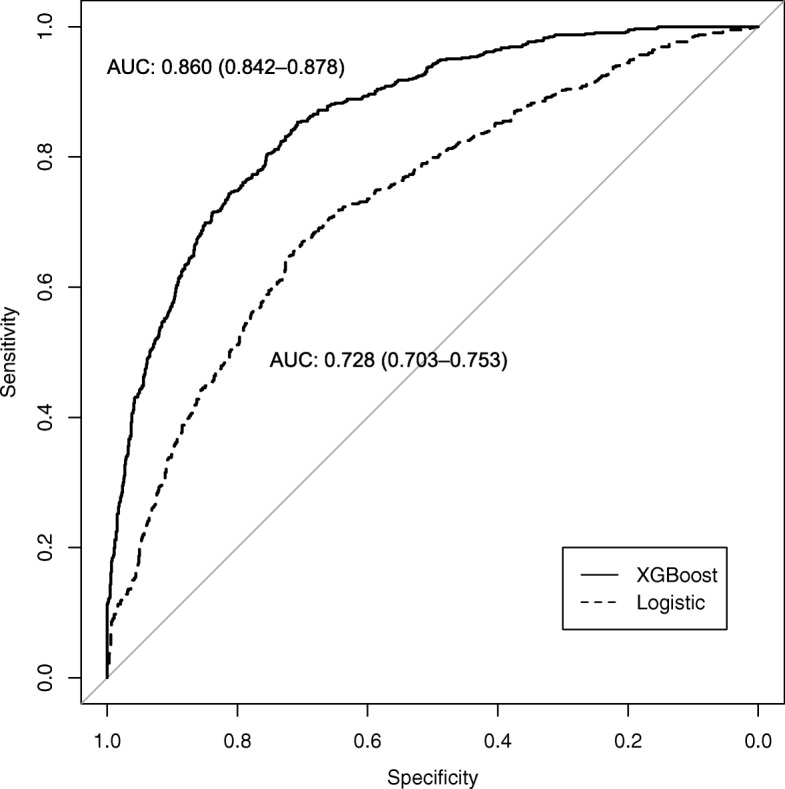
Receiver operating characteristic curve for estimating the discrimination of the logistic regression model and XGBoost model

Model discrimination was assessed using the area under receiver operating characteristic curve (AU-ROC). The XGBoost had a significantly greater AU-ROC than the logistic regression model (AU-ROC, 0.860; 95% CI, 0.842 to 0.878 vs. 0.728; 95% CI, 0.703 to 0.753, respectively; Fig. 5). Table 3 describes the classification or confusion matrix for the two models in identifying the VR and VU status.

**Table 3:** Classification matrix for the XGBoost and logistic regression models in the out-of-sample validation cohort

	XGBoost		Stepwise Logistic regression	
	Observed		Observed	
	Non-responsive	Responsive	Non-responsive	Responsive
Predicted				
Non-responsive	846	180	737	228
Responsive	177	467	286	419

Correct classification (accuracy) of volume responsiveness for the XGBoost and the logistic models were 0.79 (95% CI, 0.77–0.81) and 0.69 (95% CI, 0.67–0.71), respectively

## Discussion

In this hypothesis-generating study, we showed that some clinical factors were more likely to be associated with VR-AKI than VU-AKI. Using advanced machine learning techniques, we could identify some important clinical factors associated with VR-AKI such as age, urinary creatinine concentration, maximum BUN concentration, and albumin. These results have some implications and require further consideration.

First, an ability to accurately identify volume responsiveness in critically ill patients with AKI is clinically important to avoid both hypervolemia and hypovolemia. Currently, there is a lack of a reliable tool to distinguish between VR and VU AKI at an early stage. In this study, we showed that sophisticated machine learning techniques such as the XGBoost modeling can enrich the amount of information we can obtain from analyzing a database and allow us to develop and validate a better-performing predictive model compared to the conventional logistic regression technique. The potential usefulness of the model is that it can help to stratify oliguria patients immediately after ICU admission. As a result, large volume fluid can be more accurately given to patients who are very likely to respond to fluid challenge. There is evidence that fluid overload can result in organ dysfunctions, prolonged mechanical ventilation, and even death [19–21]. Thus, it is of vital importance to identify patients who will benefit from fluid resuscitation. However, the present study cannot provide a higher level of evidence on the effectiveness of the XGBoosting model. Future randomized controlled trials comparing the treatments with and without the prediction model are warranted to explore the effectiveness.

Second, our results showed that urinary creatinine was potentially useful to differentiate between patients in AKI who were VR and VU. Probably, patients with higher serum creatinine may also have higher excretion of creatinine to the urine. Since the former is a biomarker of kidney injury (e.g., higher serum creatinine was associated with higher risk of intrinsic injury), the latter is also associated with increased risk of VU-AKI. The utility of urinary biochemistry to predict AKI outcome has been controversial in the literature. Although urinary biomarkers such as creatinine and fractional excretion of electrolytes were significantly different between VR and VU groups in some animal and human studies [10], there are also studies showing that urinary biochemistry may not be useful in differentiating between VR and VU AKI [22–25]. In our study, we could not analyze the ability of urinary sodium and potassium to differentiate between VR and VU AKI because a large proportion (> 70%) of patients did not have this data.

Third, we found that patients with AKI and oliguria after elective surgery were more likely to respond to fluid challenges in univariate analysis. Patients who underwent elective surgery are generally in better clinical condition than patients requiring urgent surgery or with a medical emergency. Postoperative oliguria can be explained by hypovolemia due to intraoperative and postoperative insensible fluid loss. As such, they will be more likely to benefit from a larger amount of fluid after major surgery. However, the association of elective surgery with volume responsiveness disappeared after adjusting for other physiological variables, indicating that hypovolemia can be represented by these variables such as systolic blood pressure, heart rate, and hematocrit. We found that an increased hematocrit within the first 24 h of ICU admission was also an independent predictor of VR (in both the logistic and XGBoost models). This result could be explained by the fact that hematocrit has a direct relationship with the intravascular plasma volume and a higher hematocrit may suggest a relative hypovolemic state [26]. Conversely, a higher serum creatinine on ICU admission might indicate established renal intrinsic damage, which is more likely to be unresponsive to fluid challenges.

This study has some strengths and weaknesses. The XGBoost modeling is a novel technique that has not been widely adopted in critical care research. The XGBoost algorithm has been successfully used in some complex scenarios such as the prediction of the failure of the treatment for parapneumonic empyema [18], in which the predictive accuracy of the XGBoost model was significantly better than a generalized linear model. This is not surprising because the XGBoost model is an ensemble of weak prediction trees, which is able to capture complex relationships in data without the need for high-order interactions and non-linear functions to be explicitly specified [27]. Furthermore, this technique is well designed to prevent overfitting by cross-validation and regularization [17]. Our results suggest that this technique has the potential to improve the power of critical care epidemiological studies in the future. Nonetheless, this was a hypothesis-generating study, and external validation of our model is essential to confirm its utility. A limitation of this study is that we did not have data on the indications for large volume resuscitation. The study was not a designed clinical trial that the indications for large volume loading could be prespecified. However, we have randomly selected 30 cases and found that patients receiving > 5 l fluid during a 6-h period were those with indications for fluid loading in order to increase the urine output. Furthermore, the overall population had low blood pressure (mean SBP, 85 mmHg), elevated heart rate (mean, > 100/min), and lactate (mean, 3 mmol/l) in our study, which were consistent with the indications for fluid loading (Table 1). Clinicians might have given their patients fluid for a variety of different reasons such as hypotension or elevated heart rate in addition to any attempt to improve oliguria. The study only explored the short-term effect of large volume intake, other long-term outcomes such as persistent AKI, organ-failure free days, and mortality were not investigated.

## Conclusion

In conclusion, this hypothesis-generating study showed that some clinical factors were more likely to be associated with VR-AKI than VU-AKI. The XGBoost modeling technique could identify the predictors of VR-AKI that were not apparent using logistic regression, resulting in a better-performing predictive model to identify patients with VR-AKI. Further epidemiological studies using advanced machine learning techniques to validate our results will help us to identify the most suitable patients to be included in clinical trials assessing the benefits of fluid therapy in AKI.

## Abbreviations

AKI: Acute kidney injury
BUN: Blood urea nitrogen
KDIGO: Kidney Disease: Improving Global Outcomes
MIMIC: Medical Information Mart for Intensive Care
TRIPOD: Transparent Reporting of a multivariable prediction model for Individual Prognosis Or Diagnosis
VR: Volume responsive
VU: Volume unresponsive
WBC: White blood cell count
XGBoost: Extreme gradient boosting
